# Novel Therapies and Approaches to Relapsed/Refractory HL Beyond Chemotherapy

**DOI:** 10.3390/cancers11030421

**Published:** 2019-03-25

**Authors:** Alain Antoine Mina, Chetan Vakkalagadda, Barbara Pro

**Affiliations:** 1 Division of Hematology/Oncology, Northwestern University Feinberg School of Medicine Chicago Illinois, Chicago, IL 60611, USA; alain.mina@northwestern.edu; 2 Department of Internal Medicine, Northwestern University Feinberg School of Medicine Chicago Illinois, Chicago, IL 60611, USA; chetan.vakkalagadda@northwestern.edu

**Keywords:** targeted therapy, relapsed disease, refractory disease, classical Hodgkin lymphoma, salvage therapy

## Abstract

Although Hodgkin lymphoma (HL) is highly curable with first-line therapy, relapses occur in approximately 10–20% of patients with early stage disease and 30–40% of patients with advanced stage disease. The standard approach for relapsed or refractory disease is salvage therapy, followed by consolidation with high dose therapy and autologous stem cell transplant (ASCT). Patients who achieve a complete response to salvage therapy prior to ASCT have better outcomes, thus recent studies have focused on incorporating newer agents in this setting. Major challenges in the management of relapsed patients remain how to choose and sequence the many salvage therapies that are currently available and how to best incorporate novel agents in the current treatment paradigms. In this article, we will summarize the most recent advances in the management of patients with recurrent HL and will mainly focus on the role of new agents approved and under investigation. Aside from brentuximab vedotin and checkpoint inhibitors, other novel agents and therapies are showing promising early results. However, at least with some of the newest targeted strategies, it is important to recognize that we are facing new challenges in terms of toxicities, which require very close monitoring and education of both the patient and treating physician.

## 1. Introduction

Hodgkin Lymphoma is the most common lymphoma among children and young adults in the Western world [1]. The current cure rate with traditional, combined modality approaches, is anywhere between 70 to 80%, which is a very high cure rate for an aggressive lymphoma [2]. Among patients who are cured, significant short and long-term treatment related toxicities remain a significant concern, even in the current era. In addition, 20 to 30% of patients will experience disease that relapses or is refractory to conventional chemotherapy. The current standard of care for the treatment of patients with relapsed or refractory disease is salvage chemotherapy followed by autologous stem cell transplantation (ASCT), which can result in a cure rate of approximately 50% [3,4,5]. The chance of obtaining a long-term remission increases if, prior to transplant, a patient has a complete response, making the choice of salvage chemotherapy extremely important. 

The outcomes indicate that, while salvage chemotherapy followed by ASCT is the standard of care, there are still several flaws with the current approach. Given the small number of patients in studies and the different comorbidities among patients, it is unclear which particular salvage chemotherapy may be most appropriate for a given patient. Different salvage combination regimens all show complete remission rates of 20 to 60% in small studies, and there currently exist no trials that compare any of these regimens head to head [3,4,5,6,7,8]. Moreover, no two patients with relapsed or refractory disease are the same. Chemosensitivity to induction therapy, the duration of the initial remission (if any), degree of relapsed disease, age of the patient, and patient comorbidities all play a role in the eligibility and response of a given patient to a regimen [9]. This leaves the oncological community with a pressing need for new treatment modalities and strategies that would be increasingly individualized, not only to patients, but also the unique biological features of the disease. Fortunately, over the last several years, numerous advances have been made in our understanding of the biology of Hodgkin lymphoma and subsequently in our treatment. We now know that the cell surface receptor CD30 is highly expressed on Reed–Sternberg cells, making it an ideal candidate for targeted therapy. Brentuximab vedotin (BV), which is an antibody-drug conjugate that combines a monoclonal antibody targeting CD30 with the antimicrotubulin agent monomethyl-auristatin E, has been shown to have significant single agent activity in patients with relapsed/refractory disease. In addition to BV, inhibitors of the programmed death-1 (PD-1)/PD-ligand-1 (PD-L1) pathway are highly effective in the treatment of patients with relapsed/refractory disease. The upregulation of PD-L1 and PD-L2 has been identified as an important mechanism through which Reed–Sternberg cells suppress T-cell function. Furthermore, there is a unique genetic sensitivity to the blockade of this pathway, as PD-L1 is almost always expressed on the malignant cells through genetic alterations of the short arm of chromosome 9 (9p24.1), which ranges from polysomy, copy number gain, and amplifications [10]. These treatment options represent a novel less toxic approach to treatment, which may be more appropriate than the current standard of care for selected patients with relapsed/refractory disease. These treatments have been combined with each other or with other regimens in various trials as well and they have yielded promising outcomes. Additional novel agents have been explored, albeit in smaller studies, and have thus far shown promise. These therapies include everolimus, lenalidomide, panobinostat, chimeric antigen receptor T-cells, and the CTLA-4 inhibitor ipilimumab. 

This review will explore the novel treatment options available for patients with relapsed and refractory Hodgkin lymphoma—those approved thus far, and those currently under evaluation. 

## 2. Brentuximab Vedotin

Classical Hodgkin Lymphoma (cHL) is characterized by the presence of malignant Hodgkin and Reed Sternberg (HRS) cells that express the CD30 antigen, a transmembrane glycoprotein that belongs to the tumor necrosis factor receptor super-family [11]. This expression is unique and limited, making it an ideal target for therapy [12]. Over the past two decades, several studies have evaluated the safety and efficacy of different monoclonal antibodies that target CD30 in patients with relapsed HL. Early clinical trials using naked monoclonal antibodies demonstrated an excellent safety profile, but limited clinical activity [13,14]. 

Brentuximab vedotin (BV) is an antibody-drug conjugate that was developed by conjugating the chimeric anti-CD30 monoclonal antibody to the tubulin toxin monomethyl auristatin E (MMAE). Brentuximab binds to the CD30 receptor, is internalized, and then processed, leading to the release of MMAE into the cytoplasm, interruption of microtubule polymerization, and subsequent cell death [15]. In addition to direct cytotoxic activity, mechanisms that may contribute to the antitumor activity of BV include antibody-dependent cellular phagocytosis, immunogenic cell death, and very important, the bystander effect. 

A pivotal Phase II study enrolled 102 patients with relapsed/refractory HL after ASCT. Patients received brentuximab vedotin 1.8 mg/kg every three weeks for up to 16 cycles. The overall response rate (ORR) was 75% and it included an impressive 34% complete remission (CR) rate. Responses were rapid, with a median time to response of 5.7 weeks and time to achieving complete remission of 12 weeks. It is worth noting that the patients’ population that was enrolled in this study had a very poor prognosis, having received a median of 3.5 (range 1–13) prior regimens, including combination chemotherapy and ABMT. The most common treatment-related side effects were peripheral neuropathy (42%), nausea (35%), and fatigue (34%). Long-term five-years follow up showed durable remissions in 38% of patients who achieve CR [16]. It should be noted that, in addition to peripheral neuropathy, pancreatitis is a serious and potentially fatal adverse event, which was previously unrecognized. After a fatal case of pancreatitis in a patient that was treated with BV in a clinical trial, an additional fatal case and six non-fatal cases of pancreatitis were identified [17]. 

Given the promising results and the favorable safety profile that was observed with BV following transplant, a phase 3 double-blinded randomized trial (AETHERA), examined the role of BV as consolidation therapy post-autologous transplant in 329 patients. Patients with high risk features, such as refractory disease, relapse within a year of initial treatment, or extranodal disease, received 1.8 mg/kg of BV every three weeks for up to 16 cycles following transplant. Progression free survival (PFS) was evaluated by an independent review and it was significantly improved in subjects who received treatment when compared to the placebo group (hazard ratio (HR) 0.57, 95% CI 0.40–0.81; *p* = 0.0013). In fact, two-year PFS in the BV group was 65% when compared to 45% in the placebo group [18]. 

Recent data suggest that introducing BV as salvage therapy prior to transplant is feasible and beneficial. A multicenter Phase II study evaluating BV as a second line therapy prior to ASCT demonstrated an ORR of 68%. PET negativity was observed in 35% of patients who directly proceeded to ASCT without the need for salvage chemotherapy. This study further emphasized BV as a safe option and a reasonably effective bridging agent to transplant [19]. Similarly, Moskowitz and colleagues demonstrated the safety and activity of BV when used as single agent prior to ASCT. They reported a PET-negative CR rate of 27% after two cycles of therapy and these patients proceeded directly to ASCT. Patients with PET-positive disease received two courses of augmented ICE, yielding a 76% CR rate for the patients that were treated on this sequential combination [20]. To improve the response rate of chemotherapy-based salvage regimens, the addition of BV to standard chemotherapy has been evaluated in multiple clinical trials. Bendamustine is a bifunctional agent, being chemically related to the alkylating agent chlorambucil. It carries an alkylating group and a benzimidazole ring that acts as a purine analog [21]. Due to its minimal cross reactivity with other alkylating agents, bendamustine became an attractive option for combination with other alkylating agents as well as a monotherapy in the relapsed setting [21]. In a phase II study, 36 patients with relapsed/refractory cHL received bendamustine 120 mg/m^2^ as a 30-min infusion on days 1 and 2 every 28 days. The overall response rate was 53%, including 33% of patients achieving complete remission, but the median duration of response was only five months [22]. 

Given the non-overlapping toxicities of BV and bendamustine, a phase 1/2 single arm trial was designed to evaluate the efficacy and safety of these two agents in patients with refractory or first relapse cHL. The combination resulted in an ORR of 93% with an impressive CR rate of 74%, thus being significantly higher than either agent alone. The two-year progression free survival was 70% (95% CI, 50.6–82.7%) in patients who underwent transplant and 63% (95% CI, 45.7–75.6%) in the overall study population. This was a pivotal study in establishing a role for this combination, as most subjects were able to proceed to transplant after only two cycles [23].

Other trials have explored the use of BV in combination with multi-agent chemotherapy regimens. BV plus ESHAP (etoposide, solumedrol, high-dose cytarabine, and platinum) was evaluated in a Phase II clinical trial by Garcia-Sanz and colleagues. A total of 66 patients were treated with this combination and the reported ORR was 96%, with a CR rate of 70%. Additional studies, albeit in a small number of patients, have shown the feasibilty and activity of BV, in combination with other regimens, like ICE (ifosfamide, carboplatin, etoposide) and DHAP (dexamethasone, cytarabine, cisplatin) [20,24].

However, in general, combinations with multiagent chemotherapy regimens are associated with significant myelosuppression, may require hospitalization, and if we want to aim to decrease toxicity while maintaining efficacy, sequential strategies PET-guided, represent, in our opinion, the best approach. 

## 3. Checkpoint Inhibitors

Hodgkin lymphoma has a unique biology with rare HRS cells that are surrounded by an overwhelming number of immune cells. Recent data have elucidated the key role of the cross-talk between the malignant cells and the microenvironment. Perhaps one major breakthrough in HL was the discovery of 9p24.1 amplification, which leads to increased PD-1 ligands (PD-L1 and PD-L2) expression by HRS cells [10,25]. PD-L1 has also been shown to be significantly overexpressed in inflammatory immune cells that surround the cHL tumor [26]. Furthermore, Epstein-Barr virus (EBV) infection, which is commonly seen in HL, can also result in the upregulation of PDL-1. Thus, the PD1-PD1 ligand interaction provides a unique target for therapy in HL. A number of recent clinical trials have explored the safety and efficacy of immunotherapy, in particular, with checkpoint inhibitors in patients with recurrent/refractory disease, and they have shown impressive clinical results. Nivolumab and pembrolizumab are both anti-PD-1 monoclonal antibodies that have significant activity in relapsed/refractory HL. The efficacy of nivolumab in this setting was first tested in a phase I multicenter trial, in which 23 patients with relapsed/refractory cHL received 3 mg/kg of nivolumab every two weeks until disease progression or for a maximum of two years of therapy. The majority of patients in this trial had previously failed an ABMT and most had also received brentuximab vedotin. The ORR was 87% with complete responses being seen in four patients and partial responses in 16. The progression-free survival at 24 weeks was 86% [27]. CheckMate 205 was a phase II multicenter multicohort single arm trial where patients with relapsed/refractory cHL were enrolled into one of three cohorts: those who never received BV (*n* = 63), patients who received BV after autologous transplant (*n* = 80), and those who received BV before or/and after transplant. Median follow up for all three cohorts was 18 months and the ORR was 69%, with a PR rate of 53%. Responses were durable, with a median duration of 16.6 months [28,29]. Building upon the activity of BV and checkpoint inhibitors in relapsed/refractory disease, a phase 1/2 multicenter study was conducted, in which the patients were treated with a combination of BV and nivolumab as initial salvage therapy prior to ABMT. Patients received 1.8 mg/kg of BV on day 1 and 3.0 mg/kg of nivolumab on day 8 of a three-week cycle for a total of four cycles (with BV and nivolumab both being administered on day 1 in cycles 2 to 4). Most patients (98%) had adverse events, but these were mostly grade 1 or 2, with the most common side effects being nausea (49%), fatigue (41%), and infusion related reactions (44%). The ORR was 82% (95% CI, 70–91%), and the complete response rate was 61%, thus being significantly higher [30]. In fact, these results are comparable to those that were observed with BV, followed by salvage combination chemotherapy, rendering the combination BV/nivolumab an excellent alternative to standard chemotherapy and a viable initial salvage option in relapsed/refractory disease. However, a longer follow-up and a confirmatory study are needed to confirm activity and safety. Similar efficacy was seen in clinical studies evaluating the PD1 antibody pembrolizumab. A phase Ib study (KEYNOTE-013) in 31 patients who failed prior BV demonstrated a 65% ORR with 16% of patients achieving CR [31]. A larger phase II study (KEYNOTE-087) evaluated the safety and efficacy of pembrolizumab in relapsed/refractory disease and included patients who relapsed after BV. The overall response rate was 69%, with a complete response rate of 22%, and 31% of responses lasting for more than six months [32]. 

Both nivolumab and pembrolizumab are relatively well tolerated, but due to immune activation, adverse events can occur with these drugs. Thus, it is important to recognize that immune–related toxicities are unique to this group of agents and they include pneumonitis, colitis, hepatitis, skin toxicities, and endocrinopathies, among others. 

While the results with PD-1 blockade have been very encouraging, most of the responses are partial, and not all of the patients derive benefit. Besides combination approaches with chemotherapy and brentuximab vedotin, studies are also exploring combination with other immune activating agents. The combination of CTLA-4 and PD-1 blockade has shown superior efficacy in preclinical studies and solid tumor malignancies. Nivolumab has been combined with the anti-CTL4 antibody ipilimumab in patient with hematologic malignancies, including 31 patients with cHL. With a median follow-up of 11.4 months, 74% of the patients responded, thus the results were not significantly better than nivolumab alone [33].

## 4. Everolimus

Another pathway that plays a crucial role in tumorigenesis is the mTOR pathway (phosphatidylinositol 3-kinase (PI3K)/Akt/mammalian target of rapamycin), which has been shown to be dysregulated in cHL [34,35]. Everolimus is an mTOR inhibitor a rapamycin derivative with potent anti-proliferative properties that was shown in preclinical trials to be a potent inhibitor of the HL cells [36]. A phase II trial was designed to study the safety profile and efficacy of single agent everolimus in relapsed/refractory cHL. Nineteen patients with relapsed/refractory disease, who were ineligible or had failed stem cell transplant, received 10 mg of oral everolimus daily. The majority of patients had received multiple prior lines of therapy, with a median number of 6 and 84% had undergone an autologous stem cell transplant prior to trial enrollment. In this study, ORR was 47% (95% CI 24–71%), with eight patients achieving PR and 1 patient a CR. The median time to progression was 7.2 months, and four responders remained progression free at 12 months. The treatment was overall well tolerated, and the main toxicity was reversible myelosuppression [37]. Another phase 2 open-label trial enrolled 57 patients and showed similar results with an ORR of 45.6% (95% CI 32.4–59.3%) and median progression free survival of eight months (95% CI 5.1–11.0 months). However, the majority of responses were partial, with only 8.8% of the patients achieving complete response [38]. 

## 5. Histone Deacetylase Inhibitors: Panobinostat and Mocetinostat

Histone deacetylase enzymes (DACs) have been shown to play a major regulatory role in a number of cellular functions comprising angiogenesis, cell-cycle progression, and immune function. This led to their considerations as potential targets in cancer treatment [39]. In particular, multiple clinical studies have shown a promising role for HDAC inhibitors in relapsed cHL [40], though their exact mechanism of action is still unknown. Several inhibitors of these enzymes have been developed, some of which are selective of Class I enzymes, such as mocetinostat and entinostat, and others are less selective and are thus called pan-DAC inhibitors acting on enzymes that belong to Class I and II, such as vorinostat and panobinostat [41]. A phase II study was conducted to explore the safety and efficacy of panobinostat in relapsed/refractory HL. In this study, 129 patients received 40 mg of panobinostat orally three times weekly. The objective response rate was modest at 27%, 23% of patients achieved partial remission, while only 4% achieved complete response. Progression free survival was 6.1 months, but it is worth noting that 74% of the patient achieved a reduction in their tumor burden and the estimated one-year overall survival was 78% [42].

Preclinical trials have demonstrated synergy between histone deacetylase inhibitors and PI3K/AKT/mTOR pathway inhibitors, which led to efforts in exploring this combination. A phase I trial was conducted, in which 14 patients with relapsed/refractory cHL were enrolled and received a combination of panobinostat and everolimus. The ORR was 43%, with 15% of patients achieving a complete response [43]. However, the combination was associated with significant thrombocytopenia requiring dose interruptions. Similarly, a phase 1/2 trial using panobinostat in combination with ICE chemotherapy (P-ICE) showed an impressive complete response rate of 82%, but significant myelosuppression [44]. 

Another HDAC inhibitor with an established anti-neoplastic activity, including an anti-proliferative activity on HL-cells is mocetinostat, which is thought to act by inducing an upregulation of the p21 and caspase pathway and a downregulation of STAT6 [45]. Fifty-one patients were treated in a phase 2 multicenter trial and they received either 110 mg or 85 mg. The initial 23 patients received 110 mg of the oral drug three times weekly, but 85 mg was selected as the optimal dose because of treatment-related toxicities. Two patients had a complete response (both in the 110 mg cohort), 12 had partial responses (six patients in each cohort), and one patient had durable stable disease. In addition, tumor reduction was observed in 34 of 42 (81%) patients who completed at least two cycles of therapy. Although toxicity of mocetinostat was generally tolerable, four patients, all in the 110 mg cohort, died during the study, of which two might have been related to treatment [45]. 

## 6. Lenalidomide

Immunomodulatory agents (ImiDs), including lenalidomide have multiple mechanisms of actions that include anti-angiogeneis properties, and the modulation of the tumor microenvironment [46]. Lenalidomide monotherapy has shown efficacy and tolerability in a wide spectrum of neoplasms, especially in a relapse setting and has been approved by the FDA for relapsed multiple myeloma as well as 5q-myelodysplastic syndrome [46]. Lenalidomide also has shown significant activity in B-cell malignancies [47]. Lenalidomide has been tested in a phase II clinical trial of patients with relapsed/refractory disease. In this study of 38 heavily pretreated patients (median number of prior therapies of 4), the ORR was 19% and the CR rate was 3% [46]. Thus, lenalidomide has single agent that has a very modest activity, and combinations with chemotherapy or other novel agents may enhance its activity. On the basis of this single-agent activity of lenalidomide, as well as panobinostat, a recent phase I/II trial explored this combination in 24 patients with relapsed/refractory disease. The ORR was modest 16.7% (2 CR and 2 PR), lower than the ORR with either drug alone, and significant side effects far outweighed the benefits of therapy [48].

## 7. JAK Inhibitors

The JAK-STAT pathway is constitutively activated in HL and it thus represents a possible target for therapy. The Janus Kinases (JAK), when activated, phosphorylates signal transducers and activators of transcription (STAT) proteins on tyrosine residues. In turn, STAT proteins translocate to the nucleus and promote cell proliferation and survival. In addition, the JAK/STAT pathway plays a role in immune evasion by HL cells. Studies thus far have evaluated JAK inhibitors as monotherapy or in in combination with agents that block other signaling pathways that are integral to cancer growth. 

SB1518, a JAK2 inhibitor, was evaluated in a phase 1 trial of 34 patients with relapsed or refractory lymphoma of any histology, except Burkitt lymphoma or CNS lymphoma. Fourteen patients had classical HL and 20 had NHL. Out of 22 patients who received the highest doses of the drug (300, 400, or 600 mg/day), 14% (3/22) had an objective partial response. All three of these patients had non-Hodgkin lymphoma. Thirty-one patients had at least one adverse reaction, with 3 patients experiencing grade 4 toxicities. Among the 14 HL patients, no objective responses were observed. The drug was overall well-tolerated with a handful of dose-limiting toxicities [49].

Ruxolitinib, an oral JAK1/2 inhibitor, has recently been studied in a phase II trial for patients with relapsed/refractory HL. Thirty-three patients with R/R HL, among which the median number of prior therapies was 5, received a median of four cycles of ruxolitinib. While the drug was well-tolerated, the ORR was disappointing at 9.4% (3/32), with all responses being partial remissions. Reasons for inability to meet the primary efficacy goal have been hypothesized to be the very heavily treated population or inadequate dose of ruxolitinib [50]. Given the limited toxicity, perhaps the JAK inhibitors are more suitable and could be more effective in combination strategies.

A recent Phase 1 trial compared monotherapy with a PI3-kinase delta inhibitor with combination therapy of both the PI3-kinase delta inhibitor and a JAK-1 inhibitor. The study evaluated 114 total patients with various relapsed or refractory lymphomas. Thirty-nine patients had classical HL, 17 received PI3K inhibitor monotherapy, and 22 received combination therapy. The median number of prior treatments was four in both the monotherapy and combination therapy groups. The drug was largely tolerable for all patients. Interestingly, the addition of the JAK-1 inhibitor to the PI3K inhibitor doubled the ORR (67% combination, 29% monotherapy) and had higher PFS (23.1 vs. 8.3 months), although no formal study comparing the two was undertaken. This ORR of 67% is comparable to that with nivolumab and brentuximab, indicating that further study of this combination is worthwhile [51].

## 8. CAR-T Cell in Relapsed/Refractory HL

The enhancement of T cell specificity by combining it to a chimeric antigen receptor (CAR) molecule has been a major development in cancer therapeutics. CAR-T cell therapy has shown efficacy in various lymphoid malignancies [51,52,53,54], but clinical experience is more abundant in acute lymphoblastic leukemia and large B cell lymphoma. HRS cells overexpress the CD30 molecule and T lymphocytes that are redirected to eliminate CD30^+^ tumor cells through the expression of a chimeric antigen receptor (CAR) specifically binding the CD30 molecule have the potential to generate a sustained antitumor effect.

Although data is limited, and not yet mature CAR-T cell therapy, appears to be a feasible and promising approach for HL. A phase I dose escalation study treated nine patients with lymphoma, seven of which had relapsed or refractory cHL, with autologous T-cells genetically altered via a retroviral vector to express CD30 [55]. The patients were very heavily pretreated, with at least three prior lines of therapy and received three dose levels without a conditioning regimen. Two patients achieved a complete remission, long lasting, and three had stable disease, while the remainder experienced progression. Treatment was well tolerated at all doses [55]. Another recent phase I study evaluated 18 patients with relapsed or refractory lymphoma; 17 patients had HL, and one patient had cutaneous anaplastic large cell lymphoma. All of the patients had undergone at least two prior treatment regimens. Patients were pretreated with one of three ablative regimens and then received infusions of CAR-T cells with imaging, following four weeks of therapy to assess the response. Seven patients achieved partial response and six had stable disease. The infusion was generally well-tolerated: 2/18 patients experienced grade 3 or 4 toxicities [56]. A recent study that was presented at the annual meeting of the American Society of Hematology showed that the choice of conditioning regimen impacts T cell expansion and persistence, and therefore it could improve efficacy [57]. Another interesting approach is the infusion of engineered T-cells directed to the EBV latency-associated antigens [58].

These and other ongoing studies suggest that there is promise for CAR-T cell therapy in relapsed or refractory HL. 

## 9. Conclusions

The standard approach for relapsed or refractory HL following frontline treatment failure is salvage therapy, followed by consolidation with high-dose therapy and autologous stem cell transplant. Chemotherapy has been the traditional backbone of salvage therapy, however none of the regimens have been directly compared and there is no single standard of care regimen. HL has a unique biology and multiple studies have explored the use of novel agents in the setting of relapsed/refractory disease (Summarized in Table 1). These emerging novel therapies represent a new frontier for this patients’ population and increase the possibility of long-term remissions. 

Brentuximab vedotin and PD-1 targeting antibodies have thus far shown the most impressive single agent activity in heavily pretreated patients, and with the approval of these agents the management of relapsed refractory HL evolved significantly. The optimal way to use these targeted agents is also evolving, as they have been incorporated much earlier in the management of this disease. In many instances, targeted therapies have already shown equivalent efficacy and superior safety profile when compared to standard chemotherapy, which underscores the need for further clinical trials that would bring forward new agents that would ideally replace chemotherapy all together. 

As we improve our understanding of the biology of cHL, it is clear that there could be other effective therapies in patients whose disease is refractory to even brentuximab or anti-PD1 agents, including other novel agents and CAR-T cell. However, larger studies and longer follow-up are needed to better assess the safety and durability of responses. 

While the treatment landscape for patients with relapsed/refractory HL has significantly changed in the last decade, many questions remain. Further studies investigating biomarkers that predict response or toxicity could help in selecting patients who can best benefit from these novel therapies. The best sequence and duration of therapy needs to be determined. Since BV and checkpoint inhibitors have been incorporated earlier on in the treatment of HL, including in the frontline setting, the results of these studies will impact management in the salvage setting. Furthermore, in the era of targeted therapy, the role of consolidation with ABMT in complete responders should also be addressed. 

## Figures and Tables

**Table 1:** Summary of different trials and novel approaches to relapsed/refractory cHL.

Agents	Author	Year of Publication	Study Charateristics	N	Results
BV	Younes et al. [59]	2010	Phase I	45	Tumor Regression in 36/42 patient
BV	Younes et al. [16]	2012	Phase II	102	ORR 75% CR 34%
BV + augICE	Moskowitz et al. [20]	2015	Phase II	46	PET neg status in 76%-
BV	Chen et al. [19]	2015	Phase II	37	ORR 68% CR 35%
BV + Bendamustine	LaCasce et al. [23]	2018	Phase I/II	55	ORR 92.5% CR 73.6%
Nivolumab	Ansell et al. [27]	2015	Phase I	23	ORR 87% CR 17%
Nivolumab	Younes et al. [28]	2016	Phase II	80	ORR 66.3% CR 9%
Nivolumab (Checkmate 205 Trial)	Armand et al. [29]	2018	Phase II	243	ORR 69% CR 16%
BV + Nivolumab	Herrera et al. [30]	2018	Phase I/II	62	ORR 82% CR 61%
Pembrolizumab	Armand et al. [31]	2016	Phase Ib	31	ORR 65% CR 16%
Pembrolizumab	Chen et al. [32]	2017	Phase II	210	ORR 69% CR 22.4%
Everolimus	Johnston et al. [38]	2018	Phase II	57	ORR 45.6% CR 8.8%
Panobinostat	Younes et al. [42]	2012	Phase II	129	ORR 27% CR 4%
Everolimus + Panobinostat	Oki et al. [43]	2013	Phase I	30	ORR 43% CR 15%
Lenalidomide	Fehniger et al. [46]	2011	Phase II	38	ORR 19% CR 2.7%
Lenalidomide + Panobinostat	Maly et al. [48]	2017	Phase I/II	24	ORR 16.7% CR 8.3%
Mocetinostat	Younes et al. [45]	2011	Phase II	51	Disease Control 29.4%
Idelalisib	Gopal et al. [60]	2017	Phase II	25	ORR 25% CR 4%
Ruxolitinib	Van DenNaste [50]	2018	Phase II	33	ORR 9.4%, CR 0%
PI3K-inhib + JAK1 inhibitor	Phillips [51]	2018	Phase I	39	ORR 67%
CAR-T Cell	Ramos [55]	2017	Phase I	7	2 CR, 3 stable disease
CAR-T Cell	Wang [56]	2017	Phase I	17	7 PR, 6 stable disease
